# The efficacy and safety of Yijinjing exercise in the adjuvant treatment of ankylosing spondylitis: A protocol of randomized controlled trial

**DOI:** 10.1097/MD.0000000000027109

**Published:** 2021-09-24

**Authors:** Yuxuan Chen, Yixiao Ma, Zhiqiang Zhang, Yaning Zhang, Jian Jia

**Affiliations:** a Linfen People's Hospital, Linfen, Shanxi Province, China; b The Second Hospital of Shanxi Medical University, Taiyuan, Shanxi Province, China.

**Keywords:** ankylosing spondylitis, protocol, randomized controlled trial, Yijinjing

## Abstract

**Background:**

Ankylosing spondylitis (AS) is a chronic systemic autoimmune disease with high disability rate. Conventional treatment regimens have long medication cycles and are associated with adverse reactions. Therapeutic exercise is also considered to be an effective treatment for AS. Evidence suggests that Yijinjing as a low-energy exercise has advantages in adjuncting AS, but there is a lack of standard clinical studies to evaluate its efficacy and safety.

**Methods:**

This is a prospective randomized controlled trial to investigate the efficacy and safety of Yijinjing in the adjuvant treatment of AS. Approved by the Clinical Research Ethics Association of our hospital, patients were randomly divided into treatment or control groups in a ratio of 1:1. The treatment group received 4-month Yijinjing training on the basis of conventional treatment, while the control group received conventional treatment and maintained their current lifestyle. The outcome indicators included: activity index, functional ability, Bath Ankylosing Spondylitis Metrology Index, adverse reaction, etc. Finally, SPASS 22.0 software was used for statistical analysis of the data.

**Discussion:**

This study evaluated the clinical efficacy of Yijinjing exercise in the adjuvant treatment of AS, and the results of our study will provide a reference for the clinical use of Yijinjing exercise as an effective complementary alternative for the treatment of AS.

## 1 Introduction

Ankylosing spondylitis (AS) is a chronic inflammatory disease of unknown cause that mainly affects the axial bones (e.g., the spine, hip joint, and shoulders).^[1,2]^ Inflammatory back pain caused by sacroiliac arthritis and spondylitis is the main feature of AS,^[1]^ with about 70% to 80% of patients. AS onset is early, about 10% to 20% of patients with AS begin to show the first symptoms before the age of 16 years.^[3,4]^ Patients with AS often experience chronic back pain, stiffness, arthritis, and terminulitis, affecting their health and quality of life. According to statistics, patients with AS need to stop working on average 15.6 years after the disease onset, most of the patients’ functional loss occurs within 10 years,^[5]^ and about 1/3 of the patients will progress to severe disability.^[6]^ The adverse outcome of AS seriously disturb the patients’ work, family life, and inter-personal relationship, causing considerable psychological distress and fear.

Non-steroidal anti-inflammatory drugs, including COX-2 inhibitors, are recommended as first-line interventions to reduce pain and stiffness. Biological disease-modifying anti-rheumatic drugs have also been shown to be effective in controlling the progression of AS.^[7]^ However, some AS patients do not respond well to drug intervention and the efficacy is not ideal.^[8]^ Currently, practice guidelines recommend a combination of drug and non-drug therapies to optimize the treatment of AS.^[9,10]^ There is evidence that exercise is effective in the treatment of AS, and the guidelines also recommend exercise as a combination intervention in combination with pharmacological interventions for the treatment of AS.^[9,11]^ Affected by the pathological characteristics of AS, AS patients are not suitable for high-intensity exercise, so low-intensity or moderate intensity physical and mental exercise is the preferred exercise program for AS patients.^[12]^

As one of the traditional Chinese exercises, Yijinjing is a moderate intensity exercise therapy based on the theory of traditional Chinese medicine. The Yijinjing exercise emphasize the combination of symmetrical physical postures, meditative mind, and breathing techniques in a harmonious manner.^[13]^ In China, Yijinjing, like Tai Chi, is believed to regulate the balance of Yin and Yang, dredge qi and blood, and prolong life. At present, it has been used in the treatment of osteoarthritis, skeletal muscle balance disorder, and other diseases. It can improve the contraction function and coordination of skeletal muscle, regulate emotions, and relieve psychological stress,^[14–16]^ and its efficacy has been shown to be complementary and alternative in some osteoarthritis diseases.^[17]^ By improving the flexibility of patients, improving the cardiovascular system, increasing muscle strength, increasing joint flexibility, and reducing joint pain, Yijinjing can have a positive impact on the activity level of AS. Although Yijinjing has advantages in the adjuvant treatment of AS, there is no randomized controlled trial (RCT) on the adjuvant treatment of AS with Yijinjing, and there is also a lack of follow-up observation on its long-term efficacy. Therefore, we intend to evaluate the efficacy and safety of Yijinjing exercise in the adjuvant treatment of AS through this RCT.

## 2 Materials and methods

### 2.1 Study design

**Figure 1:**
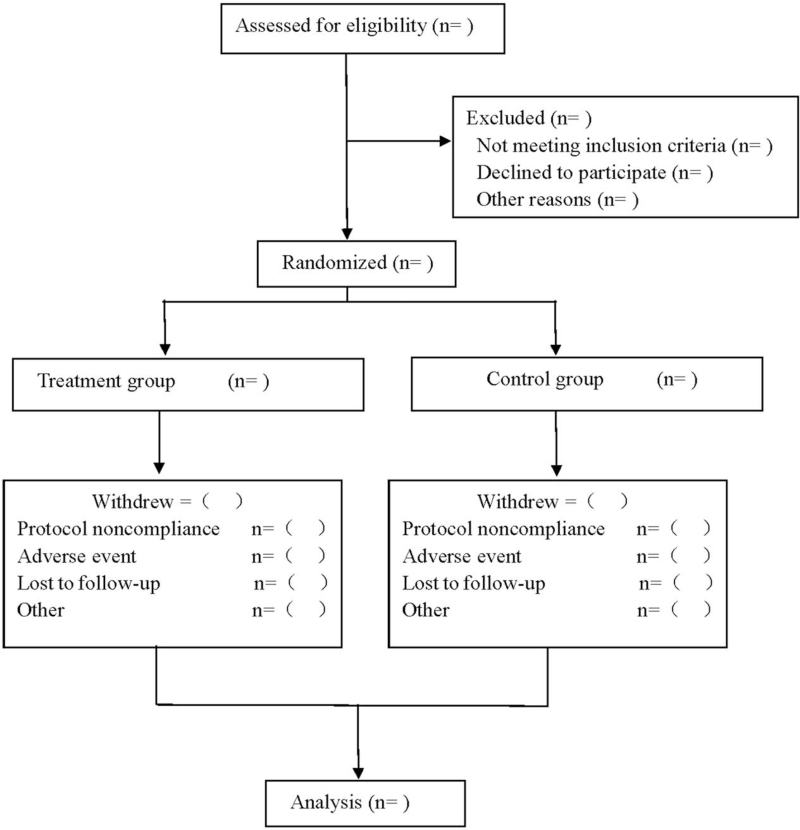
Flow diagram.

This is a prospective RCT to study the efficacy and safety of Yijinjing exercise in the adjuvant treatment of AS. This trial will follow the intervention reporting criteria for controlled acupuncture and moxibustion trials^[18]^ and comprehensive test reporting standards.^[19]^ See Figure 1 for the flow diagram.

### 2.2 Ethics and registration

The study protocol complies with the Declaration of Helsinki and has been approved by the Clinical Research Ethics Committee of our hospital. This experiment has been registered with Open Science Framework platform (registration number: DOI 10.17605/OSF.IO/VNWBC). Before randomization, all patients were required to sign an informed consent form, and they could always choose whether to proceed with the trial.

### 2.3 Sample size

The sample size was estimated based on the mean and standard deviation of Bath Ankylosing Spondylitis Disease Activity Index (BASDAI)^[20]^ scores after treatment. According to the results of the pilot study, the treatment group was 3.08 ± 1.53, and the control group was 4.19 ± 1.72. Set α = 0.025, unilateral test, β = 0.10. Calculated by PASS15.0 software, each group needed 47 participants, the estimated dropout rate was 20%, and 59 patients would be included in each group.

### 2.4 Patients

Inclusion criteria: patients meeting the diagnosis of AS (refer to the Modified New York Criteria for AS^[21]^); 18 to 60 years old, BASDAI^[20]^ ≥4; no recent (within 1 month) treatment (e.g., biologics, anti-rheumatic drugs, non-steroidal anti-inflammatory drugs, etc); not participating in any exercise program (such as yoga, Tai Chi, or regular swimming) at the time of screening; patient compliance was strong and informed consent was signed.

Exclusion criteria: participants were excluded if they met any of the following criteria: was unable to complete the entire Yijinjing exercise based on evaluation by the researchers (e.g., severely limited activity); not suitable for practicing Yijinjing exercise based on evaluation by the researchers (e.g., patients with vertebral compression fracture, severe lumbar disc herniation, severe cardiopulmonary disease); inability to provide informed consent due to mental or behavioral disorder; with the presence or history of other medical conditions that may reduce or complicate enrollment.

### 2.5 Study design

Software was used to generate random sequences, and the corresponding numbers were placed in sealed, opaque envelopes that were opened with each patient's consent to participate. Tasks were performed by a research assistant who was not involved in recruitment to ensure that assignments were hidden. Subjects were randomly divided into treatment group and control group in a ratio of 1:1. Participants who met the criteria were informed of their assignments by the project manager over the phone after the baseline measurements were completed. Due to the limitations of the intervention program, the allocation plan was known to the patients and the principal investigator during the study, but was not known to the outcome evaluators and data analysts.

### 2.6 Interventions

Both groups received routine treatment for 30 days, indometacin enteric-coated tablets (Datong Liqun Pharmaceutical Co., LTD., National drug approval number H14020511), 25 mg/time, twice/d orally; sulfasalazine (Shanghai Zhongxi 3d Pharmaceutical Co., LTD., National drug approval H31020450) 1 g/time, twice/d, oral.

(1) Treatment group: Patients underwent 40 minutes of Yijinjing exercises 3 times a week for 4 months. The training was divided into 3 parts: warm up for 5 minutes, Yijinjing exercise for 30 minutes, muscle stretching for 5 minutes. A whole set of Yijinjing exercises consists of 12 postures. Each pose would be demonstrated to the participants by a professional and the exercises would be supervised throughout the trial. The same researcher would instruct all participants to maintain a normal lifestyle, and the postures would be personalized according to their abilities.
(2) Control group: Patients were asked to maintain their current lifestyle for 4 months. Changes to existing medications were not allowed, while the use of drugs for other diseases was permitted. Participation in other sports such as yoga, Tai Chi, and gymnastics was not allowed. However, general activities such as walking, stretching, or the occasional swim were not prohibited. All adverse events were required to be recorded and reported to the investigator.

### 2.7 Evaluation criteria and curative effect judgment

(1) Primary outcomes: BASDAI^[20]^; Functional Ability (measured using the Bath Ankylosing Spondylitis Functional Index).^[22]^
(2) Secondary outcomes: Bath Ankylosing Spondylitis Metrology Index^[22]^; Chest expansion (cm); Nocturnal spinal pain; Adverse reaction: symptoms of treatment-related discomfort during treatment.

All outcome measurements were assessed at baseline and at the conclusion of the treatment period. Differences were compared between the 2 groups.

### 2.8 Data collection and management

One or 2 assistants collected and recorded all the data. Personal information about potential and registered participants would be collected, shared, and kept in a separate repository to protect confidentiality before, during, and after the trial. Access to the database would be limited to the researchers on the research team.

### 2.9 Statistical analysis

The collected data were analyzed statistically by SPSS 22.0 software. Chi-square test was used for counting data. Mean ± standard deviation ($\bar{x}$ ± S) was used for measurement data, independent sample *t* test was used for normal distribution, and Mann-Whitney *U* test was used for skewness distribution. The difference was considered statistically significant when *P* < .05.

## 3 Discussion

It is well known that exercise is very important in the treatment of AS. International guidelines recommend exercise as a treatment to improve/maintain range of motion, muscle strength, and well-being in patients with AS,^[9]^ and the International Association of Spine Joints also recommends that AS patients receive regular exercise in the spine arthritis assessment guidelines.^[22]^ For AS patients, it is important to choose a simple, easy to learn, safe, and effective exercise.

As a traditional Chinese exercise, Yijinjing was developed on the basis of the theory of traditional Chinese medicine. It harmonizes Yin and Yang, connects the body and mind, and can regulate psychological stress while improving physical discomfort.^[13]^ In practical operation, it is easy to learn and not limited by time and space. As a kind of moderate intensity exercise, it has no complex body movements and is easy to accept for most AS patients.^[23]^ Previous studies have mostly focused on chronic diseases such as Parkinson disease, stroke, osteoarthritis, cancer fatigue, etc. Therefore, our study will be the first RCT to evaluate the efficacy of Yijinjing exercise in patients with AS. We expect that Yijinjing exercise will be a safe and effective treatment for the adjuvant treatment of AS, and we expect that the results of this study will provide evidence for clinicians to choose Yijinjing exercise for the treatment of AS.

There are also some deficiencies in our study: due to the factors of treatment mode, this study could not achieve double-blind method, which may affect the results of the study. This study requires patients to participate actively, and patients’ false feedback would have a negative impact on the results. At the same time, there may be population regionalization in single-center studies.

## Author contributions

**Conceptualization:** Yuxuan Chen.

**Data curation:** Yixiao Ma, Yaning Zhang.

**Formal analysis:** Zhiqiang Zhang.

**Funding acquisition:** Jian Jia.

**Software:** Zhiqiang Zhang.

**Supervision:** Yaning Zhang.

**Writing – original draft:** Yuxuan Chen.

**Writing – review & editing:** Yixiao Ma, Yuxuan Chen.
